# The notch sign as a clue for the diagnosis of early cutaneous T-cell lymphoma

**DOI:** 10.1016/j.jdcr.2024.11.029

**Published:** 2024-12-03

**Authors:** Evelyne Harkemanne, Léo-Paul Secco, Marie Baeck

**Affiliations:** a Department of Dermatology, Cliniques universitaires Saint-Luc UCLouvain, Brussels, Belgium; b Department of Pathology, Cliniques universitaires Saint-Luc, Brussels, Belgium

**Keywords:** clinical sign cutaneous T-cell lymphoma, diagnostic aid, folliculotropic mycosis fungoides, notch sign, skin cancer

## Introduction

We report a severe case of cutaneous T-cell lymphoma (CTCL) that progressed from an early plaque stage, undiagnosed for several years, to an advanced tumor stage with fatal consequences. Nevertheless, this case of mycosis fungoides (MF) could have been diagnosed at an earlier stage thanks to the presence of the notch sign in association with other clinical diagnostic signs of MF. The notch sign refers to the sometimes sharp, well-defined, notch-like demarcations of MF plaques. Although nonspecific, this sign is an additional aid to guide clinicians toward iterative skin biopsies to unmask MF.

## Case report

**Fig 1:**
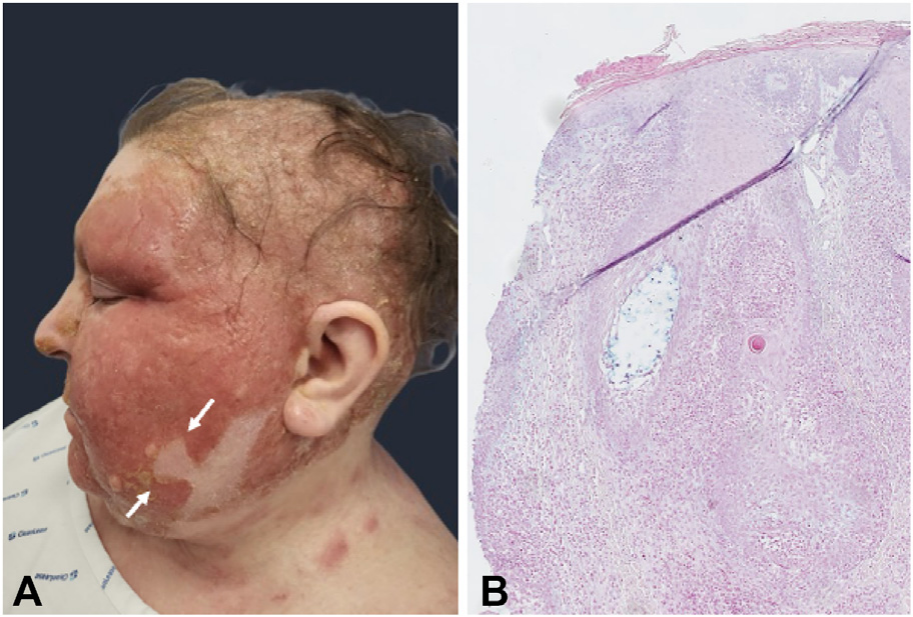
**A,** Clinical photograph of mycosis fungoides of the face in the form of infiltrative scaly patches showing well-defined “notched edges” (=notch sign; *white arrows*) associated to secondary alopecia. Misdiagnosis is very common in early cutaneous T-cell lymphoma. Therefore, the presence of the notch sign in association with other clinical diagnostic signs of mycosis fungoides is an important additional help to guide the clinician. **B,** Histological findings of the patches showed follicular invasion by atypical CD3+ T cells associated to follicular mucinosis.

A 48-year-old woman presented to the dermatology clinic with a 12-year history of pruritic erythematous rash that initiated on her limbs and progressively extended to the trunk and face, diagnosed as atopic dermatitis (AD) and treated for years with topical steroids without complete response. She had no further medical history. On examination, the clinical review of systems was normal with exception of infiltrative scaly patches with well-defined “notched edges” observed on the upper body and head (Fig 1, *A*). On the scalp, extension of the plaques led to secondary alopecia of the hair and eyebrows.

**Fig 2:**
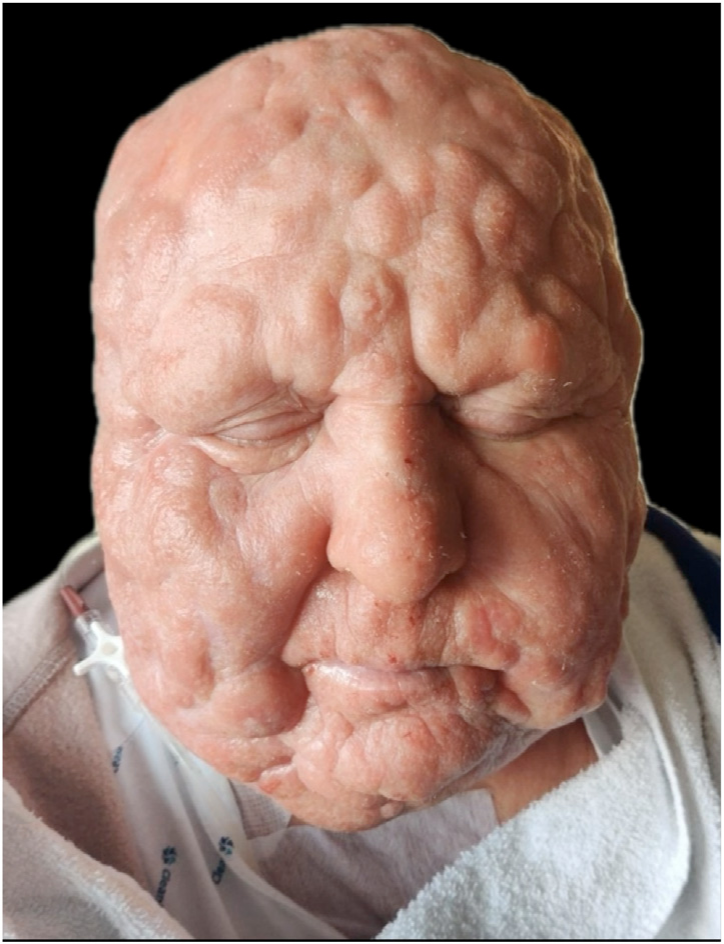
Progression of cutaneous T-cell lymphoma to tumor stage with characteristic leonine facies after failure of several lines of treatment.

Skin biopsies were performed and histopathological findings confirmed the diagnosis of CTCL in the form of folliculotropic MF (Fig 1, *B*).^1^ Additional laboratory investigations, including morphological examination and immunophenotyping of the peripheral blood, did not show the presence of circulating Sezary cells. However, positron emission computed tomography scan imaging revealed multiple hypermetabolic peripheral lymph node enlargements. Over time, the skin lesions thickened to become copper-colored tumors, giving rise to the characteristic leonine facies (Fig 2).

Several lines of treatment were administered but the disease relapsed consecutively after topical steroids, UV radiation therapy, oral methotrexate, bexarotene, and five lines of chemotherapies (mogamulizumab [CCR4 monoclonal antibody], doxorubicine, brentuximab, gemcitabine, etoposide/ifosfamide/rituximab). Unfortunately, the patient died of disseminated intravascular coagulation during the fifth line of treatment.

## Discussion

MF is the most common type of CTCL, with the folliculotropic form known for its poorer prognosis and treatment resistance.^2^ Skin-directed treatments (topical steroids, UV radiation therapy, topical chlormethine, and bexarotene) are considered the most appropriate for early-stage MF. In advanced stages, several consecutive systemic treatments are often required due to their short duration of response.^3^

Diagnosis of MF is essentially histopathological, and multiple iterative skin biopsies are often required to confirm clinical suspicion. However, given the wide spectrum of clinical presentations of MF, misdiagnosis is frequent and can lead to a delay in diagnosis of several years.^4^ In this context, when faced with aspecific dermatitis or atypical forms of known dermatitis, diagnostic signs that lead to the suspicion of MF are essential. In addition to the well-known signs, such as areas of healthy skin between lesions, secondary alopecia, and violaceous infiltrative skin patches, the presence of clearly demarcated patches showing occasional notching is a complementary sign for MF. The notch sign was first described by the *Groupe Français d’Étude des Lymphomes Cutanés* as a nonspecific but highly predictive clinical sign of early-stage MF when found on a patient’s skin.^5^ However, this sign is poorly reported in the literature, and no data on its sensitivity or specificity for MF are nowadays available. Furthermore, it does not predict disease progression.

This “dermatological” notch sign must be differentiated from the “convoluted” or “reniform” appearance of the nucleus of Sezary cells (atypical circulating CD4+ lymphocytes).^6^ While the dermatological notch sign is an early sign of MF, leukemic Sezary cells appear in the peripheral blood in patients with advanced CTCL. In addition, a radiological “notch sign” has also been reported for the noninvasive diagnosis of primary central nervous system lymphomas, with some of these lymphomas showing ring-shaped contrast enhancement on magnetic resonance imaging.^7^ Therefore, the presence of lesions with “notched” edges appears to be a criterion associated with lymphomas and is a good way to remind clinicians that the observation of a clinical notch sign should raise suspicion of MF. With current observations of lymphoid reactions/rapid development of MF associated with dupilumab in patients with AD, clinicians are urged to remain even more vigilant in differentiating MF from AD before initiating dupilumab.^8^ In this context, the notch sign, associated with adult-onset AD without personal or familial atopic medical history and with an atypical clinical presentation, is a red flag to perform skin biopsies prior to or during treatment in case of worsening skin lesions in order to reveal potential undiagnosed MF.^9^

To conclude, with the aggressiveness of CTCL increasing considerably from the tumor stage onward, the notch sign, despite being nonspecific, should be systematically sought in the case of erythematous, scaly skin patches, as it is a valuable additional aid to rule out the need for skin biopsies and avoid significant delay in diagnosis.

## Conflicts of interest

None disclosed.
